# Supplementary figures and images for: A Phase II Study on the Use of Convalescent Plasma for the Treatment of Severe COVID-19- A Propensity Score-Matched Control Analysis
Source: Microorganisms. 2021 Apr 11;9(4):806. doi: 10.3390/microorganisms9040806 (PMC8069820; doi:10.3390/microorganisms9040806)

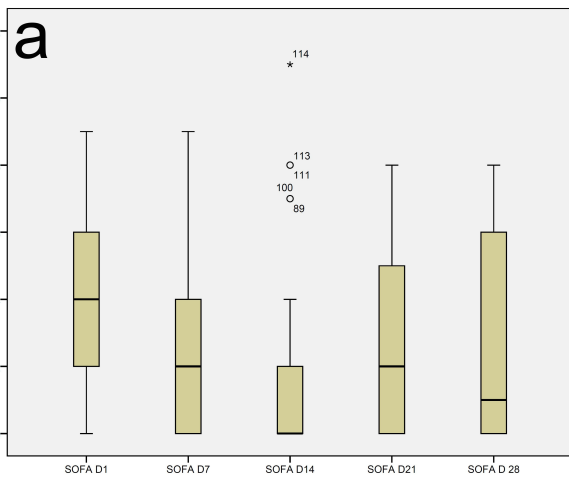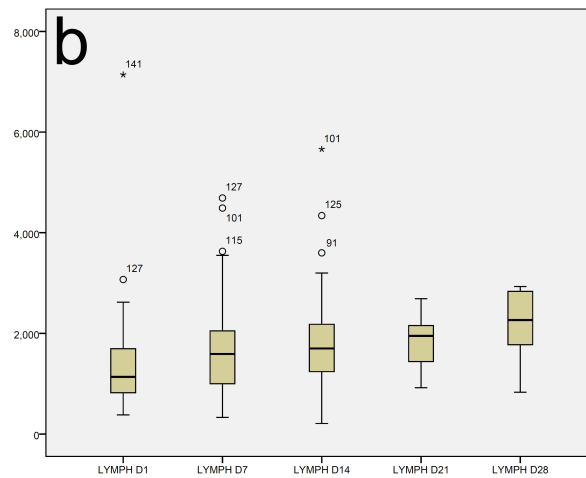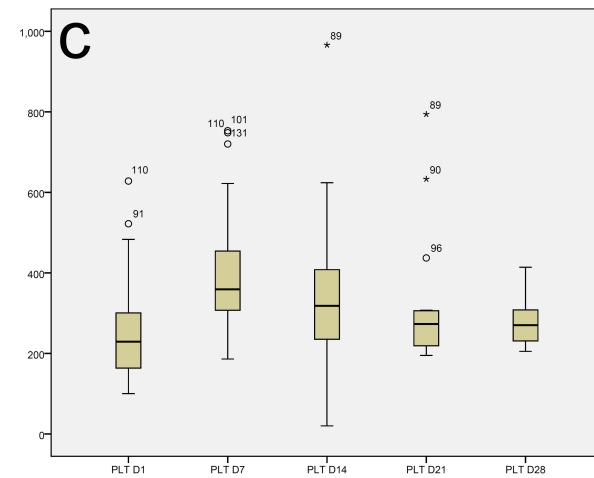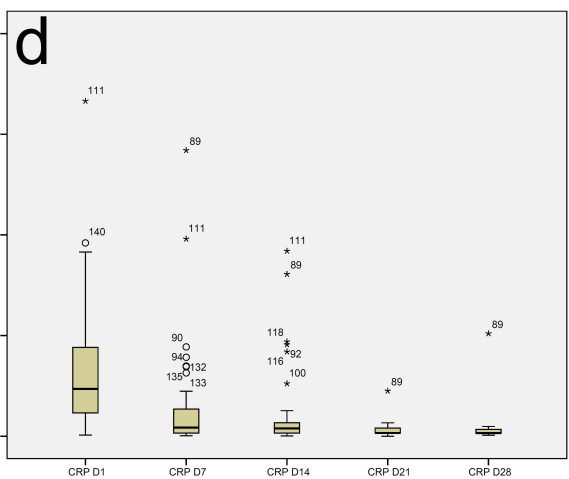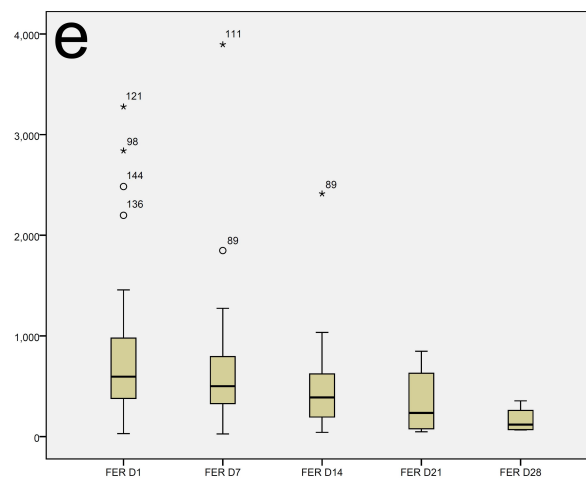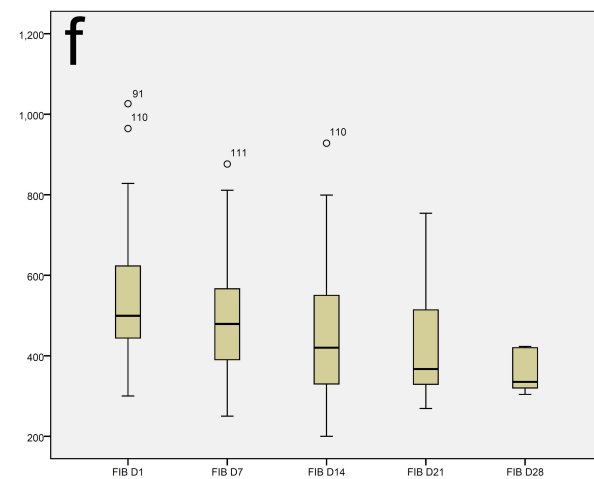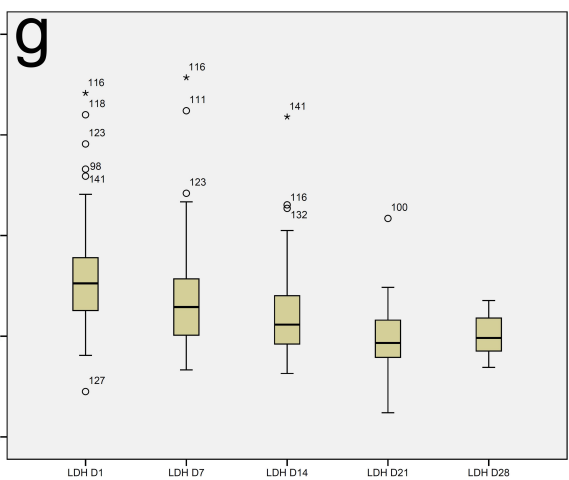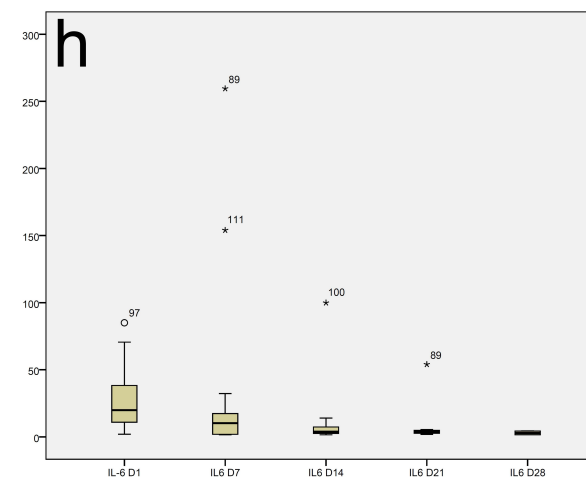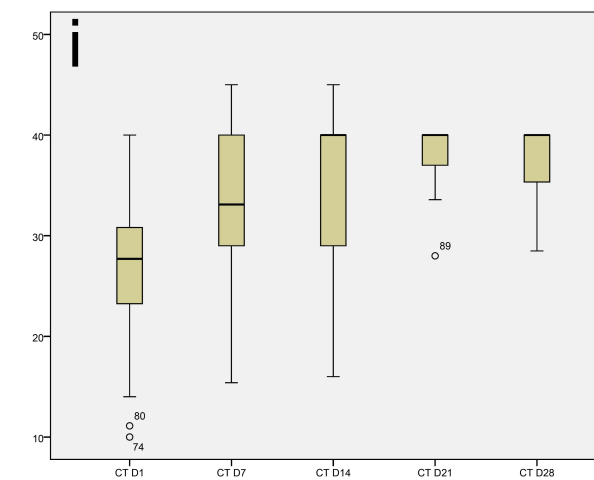

Supplement: Supplementary file 1 [file microorganisms-09-00806-s001.pdf]
